# A Clinical Case, with Comments

**Published:** 1888-11

**Authors:** Edward Bayard

**Affiliations:** New York


### A CLINICAL CASE, WITH COMMENTS.

EDWARD BAYARD, M. D., NEW YORK.

Homœopathy is capable of precision in its practice, but she ever demands a close and thorough study of symptoms of the parts affected—of the conditions under which those symptoms are manifested, of the accessory symptoms—all of which being observed and traced, lead with certainty to the nerve-cell diseased. In this way the remedy can always be given with precision, and produce a cure. Many a case has been utterly spoiled, and the cure defeated by the practitioner's not knowing or considering the duration of the action of medicine which may be overwhelming and destructive to the end for which the medicine was given.

An able and observing physician of the city of New York told me that in his practice he had a patient who had lost his smell and taste, which was a great deprivation—that he cultivated flowers and enjoyed exceedingly their perfume. He sought the advice of the doctor, who examined his case and prescribed a single dose. Shortly after the patient left the town and was absent for some time. When he returned, much to his joy, he had recovered entirely his smell. The doctor, though having done well, thought he might do better with a few more doses of his remedy. After taking the medicine the patient relapsed, and with all his skill the physician could not excite again a reaction.

I believe if he had left nature to do her work without further assistance, the cure would have been permanent.

I cite a case from my *own practice*, which goes to show that the remedy, if well selected, *must* be permitted to act, and if the reaction is not interrupted will result in a cure.

An infant, five months old, a girl, fair and fat, was taken ill at the end of December last, with great hoarseness and with high fever. Two doses of Aconite were administered by the parents, but they failed to abate the suffering. The hoarseness rapidly increasing, I was called to see the child. She was breathing rapidly with great difficulty; had a cough dry and gagging—a cough suffocative with dyspnœa, and croupy. The eyes were heavy and dull; face expressive of pain.

On the examination of the throat, white patches covered the left tonsil; the throat was so stopped that all the child's cries were smothered, and it could hardly utter a sound.

After examining the child for some time, from the totality of the symptoms I prescribed one dose of Apis $^{cm}$ (Fincke). The child lay all day in the same state, but no worse. The next morning about five o'clock she appeared alarmingly worse, the breathing being almost impossible. I saw the child at nine o'clock that morning. There was a slight improvement in the cough, and there were marked symptoms of the action of the medicine. On coughing, pain in head, which was shown by the distressed look and corrugations of the brow. The second morning the child appeared again worse about five or six o'clock; small bilious stools set in. She rallied again between eight and nine o'clock in the morning. From that time improvement was constant. The patches from the throat disappeared; the cough subsided, and in ten days the baby was able to leave her room. She never had a relapse, and lost but little flesh. She had but one dose of Apis, and no more medicine was given.

Diphtheria on the left side is Apis; suffocative cough is Apis; the aggravation in the morning is Apis; the pain in the head on coughing is Apis; and likewise the diarrhœa.

I believe if Apis had been interfered with in this acute affection, the error would have terminated the life of the child.—*Trans. I. H. A.*

